# Supplementary material for: Functional Connectivity Alterations Based on Hypometabolic Region May Predict Clinical Prognosis of Temporal Lobe Epilepsy: A Simultaneous 18F-FDG PET/fMRI Study
Source: Biology (Basel). 2022 Aug 5;11(8):1178. doi: 10.3390/biology11081178 (PMC9404714; doi:10.3390/biology11081178)
Supplement: Supplementary file 1 [file biology-11-01178-s001.zip › biology-1796983-supplementary.pdf]

Table S1. Correlations between the SUVr and GMV of hypometabolic areas in patients.

TLE	Hypometabolic areas	r	P
Left TLE	L HG	0.225	0.439
	L PHG	0.190	0.515
	L STG	-0.306	0.287
	L MTG	-0.752	0.184
	L ITG	-0.421	0.134
Right TLE	R HG	-0.118	0.641
	R PHG	-0.079	0.755
	R STG	0.154	0.541
	R MTG	-0.104	0.680
	R ITG	-0.200	0.417

TLE: temporal lobe epilepsy, L: left, R: right, STG: superior temporal gyrus, MTG: middle temporal gyrus, ITG: inferior temporal gyrus, PHG: parahippocampal gyrus, HG: hippocampal gyrus

Table S2. Correlations between epilepsy duration and FC values of brain areas showing decreased connection with structural ROI.

Brain areas with decreased FC	r	P value
Left superior frontal gyrus	-0.098	0.592
Left medial frontal gyrus	-0.247	0.172
Right medial frontal gyrus	-0.262	0.147
Right superior temporal gyrus	0.077	0.677
Left middle temporal gyrus	0.017	0.928
Right middle temporal gyrus	0.003	0.989

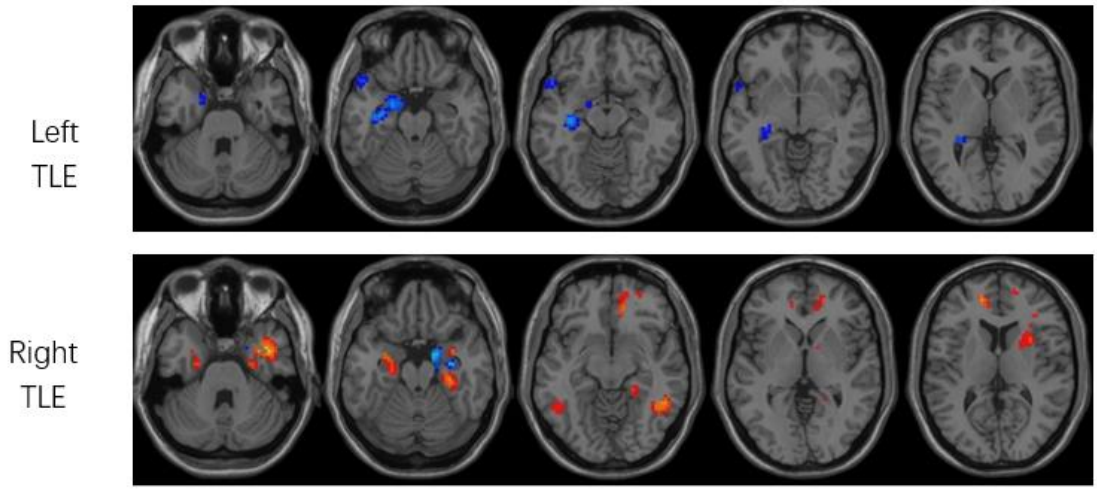

Figure S1. Comparisons of voxel-based GMV alterations between TLE patients and controls. Brain areas with hot color indicate significantly increased GMV, while brain areas with cold color indicate significantly decreased GMV (GRF corrected, $P < 0.01$). For both patients with left or right TLE, the shared area with GMV reduction was only detected in the ipsilateral hippocampus.
